# Glycoengineering of Interferon-β 1a Improves Its Biophysical and Pharmacokinetic Properties

**DOI:** 10.1371/journal.pone.0096967

**Published:** 2014-05-23

**Authors:** Kyoung Song, In-Soo Yoon, Nam Ah Kim, Dong-Hwan Kim, Jongmin Lee, Hee Jung Lee, Saehyung Lee, Sunghyun Choi, Min-Koo Choi, Ha Hyung Kim, Seong Hoon Jeong, Woo Sung Son, Dae-Duk Kim, Young Kee Shin

**Affiliations:** 1 College of Pharmacy and Research Institute of Pharmaceutical Sciences, Seoul National University, Seoul, Republic of Korea; 2 Institutes of Entrepreneurial BioConvergence, Seoul National University, Seoul, Republic of Korea; 3 Research Institute of Reference Biolabs. Inc., Seoul, Republic of Korea; 4 College of Pharmacy and Natural Medicine Research Institute, Mokpo National University, Jeonnam, Republic of Korea; 5 College of Pharmacy, Dongguk University-Seoul, Gyeonggi-do, Republic of Korea; 6 PanGen Biotech Inc., Gyeonggi-do, Republic of Korea; 7 College of Pharmacy, DanKook University, Chungnam, Republic of Korea; 8 Biotherapeutics and Glycomics Laboratory, College of Pharmacy, Chung-Ang University, Seoul, Republic of Korea; 9 College of Pharmacy, CHA University, Gyeonggi-do, Republic of Korea; George Washington University, United States of America

## Abstract

The purpose of this study was to develop a biobetter version of recombinant human interferon-β 1a (rhIFN-β 1a) to improve its biophysical properties, such as aggregation, production and stability, and pharmacokinetic properties without jeopardizing its activity. To achieve this, we introduced additional glycosylation into rhIFN-β 1a via site-directed mutagenesis. Glycoengineering of rhIFN-β 1a resulted in a new molecular entity, termed R27T, which was defined as a rhIFN-β mutein with two N-glycosylation sites at 80^th^ (original site) and at an additional 25^th^ amino acid due to a mutation of Thr for Arg at position 27^th^ of rhIFN-β 1a. Glycoengineering had no effect on rhIFN-β ligand-receptor binding, as no loss of specific activity was observed. R27T showed improved stability and had a reduced propensity for aggregation and an increased half-life. Therefore, hyperglycosylated rhIFN-β could be a biobetter version of rhIFN-β 1a with a potential for use as a drug against multiple sclerosis.

## Introduction

Multiple sclerosis (MS) is a chronic neurodegenerative disease affecting the brain and spinal cord, leading to symptoms, including blurred vision, muscle weakness, trouble with mobility and balance, cognitive and memory problems, and sensory disturbances [1]–[3]. It typically occurs between the ages of 20 to 50, is more common in women than in men, and has a variable course [3], [4]. As gold standard MS therapeutics, recombinant human interferon-β (rhIFN-β) products are widely used as a first-line treatment and have had a good long-term safety record over the last few decades [5], [6]. Although rhIFN-β is currently competitive with newer oral medicines that provide improved compliance and tolerance, it is still uncertain whether injectable treatments can be completely replaced by oral drugs, at least until oral drugs demonstrate good long-term safety records [6], [7]. This is particularly important because drug safety is one of the biggest issues in MS therapeutics, as MS is not a life-threatening disease but a life-long disease (since it occurs at an early age) [4], [6]. Therefore, the production of biobetter versions of rhIFN-β would fulfill considerable unmet needs in the MS therapy, both with respect to considerations within the pharmaceutical industry, such as biophysical stability and low production costs, and in medicine, such as fewer side effects, longer dosing intervals and route of administration.

Protein modification with polyethylene glycol (PEG) or oligosaccharide moieties is an approach often used to improve bioactive proteins, especially in regard to physical and thermal stability, increased solubility, protection against enzymatic digestion, increased circulating half-life, and in some cases, decreased immunogenicity [8]–[10]. Notably, next generation rhIFN-β therapeutics has been created using Fc fusions or PEGylation of rhIFN-β at its N- or C-terminal region, or at cysteine residues. PEGylated-Avonex is currently undergoing phase III clinical trials [11]. However, production problems with rhIFN-β 1a still remain a concern for the development of PEG-rhIFN-β 1a, because of rhIFN-β 1a aggregation, and the increased production cost associated with PEGylation.

Unlike PEGylation, glycoengineering requires no additional manipulation processes after the construction of a relevant cell line because glycosylation is a natural protein modification within mammalian cells. In addition, it is well known that glycosylation is important for the activity of rhIFN-β [12], [13]. There are two types of therapeutic rhIFN-β in clinical use: rhIFN-β 1a (Avonex and Rebif), which is produced in CHO cells and is singly glycosylated, and rhIFN-β 1b (Betaseron), which is produced in *Escherichia coli* and is not glycosylated [14]–[16]. In many reports, rhIFN-β 1a shows a higher specific activity, lower immunogenicity and decreased propensity for aggregation than rhIFN-β 1b [13], [16]. Runkel *et al*. demonstrated that this remarkable difference is due to glycosylation, in which the glycan moiety confers protein stability, solubility and biological activity. Thus, glycoengineering may be a promising approach for improving the biophysical properties of glycoproteins, such as their structural stability and pharmacokinetics [17].

Although rhIFN-β 1a is remarkably more stable and active than rhIFN-β 1b, manufacturing of rhIFN-β 1a still continues to suffer from variable levels of expression and stability in mammalian cell lines, which often results in a low product yield [18], [19]. The main challenge for developing rhIFN-β 1a is aggregation and poor stability, which can occur at very different stages in the development processes, and can result in low host viability, low productivity and the development of precipitates in solutions [19], [20].

The approach outlined in the present study used site-specific hyperglycosylation via site-directed mutagenesis, which resulted in the development of a new molecular entity, termed R27T. Site-specific hyperglycosylation of R27T was confirmed by western blot analysis, isoelectric focusing, enzyme immunoassay, PNGase treatment and quantification of sialic acid. R27T displayed superior stability, solubility, productivity and pharmacokinetic properties without loss of specific activity or alterations in ligand-receptor binding.

## Materials and Methods

### Ethics Statement

All experimental procedures and protocols for animal study had been approved by the Institutional Animal Care and Use Committee of the Seoul National University (protocol #SNU-200909-33). All efforts were made to minimize suffering of animals.

### Gene Construction, Expression and Purification of rhIFN-β 1a Glycosylation Analogs

Glycosylation analogs were constructed by performing site-direct mutagenesis via PCR on wild-type human IFN-β. The full-length gene was recovered and cloned into the pMSG expression vector (Patent#. US20040038394 A1, PanGen Biotech Inc., Gyeonggi-do, Korea). Stable transfection into CHO cells was performed using a dihydrofolate reductase selection system and selected methotrexate resistance clones were grown in methotrexate selective medium. Purification of rhIFN-β 1a and rhIFN-β mutant proteins, such as D110N, R27T and R27T-(NITV)2 was performed by PanGen Biotech Inc., in the same way. Firstly, for their purification, culture fluid containing proteins was applied to a column of blue Sepharose 6FF (GE Healthcare, Buckinghamshire, UK), which was then eluted with 35% propylene glycol-based phosphate buffer. After elution, the eluate was sequentially loaded onto CM Sepharose FF (GE Healthcare) and to C4 Reverse phase-high performance liquid chromatography (RP-HPLC) (Vydac, CA, USA). Purified proteins were concentrated and diafiltrated using a 10 kDa cut-off membrane in a Millipore Labscale Tangential Flow Filtration System (Millipore, MA, USA). Finally, gel filtration chromatography was performed on a Sephacryl 100HR column at 2.5 mL/min. An additional product, termed R27TΔGlyc, during R27T purification was also obtained from the C4 RP-HPLC purification step, which was separated from R27T in this step. R27TΔGlyc consisted of singly or double glycosylated rhIFN-β mutein at a ratio of approximately 7∶3 (data not shown). As control material, Rebif was purchased from Merck KGaA (Hesse, Germany), respectively.

### Analysis of Expressed Proteins

Purified rhIFN-β mutant samples were assessed by SDS-PAGE and western blotting using Anti-rhIFN-β antibody (R&D Systems, MN, USA) as a primary antibody. To confirm the presence of glycosylation, 1 mg/mL R27T in 20 mM sodium phosphate monobasic dihydrate, pH 7.5, was treated with PNGase F (Sigma-Aldrich, MO, USA) at 37°C. Samples were taken at different times over a period of 1 hr. Deglycosylation was monitored by SDS-PAGE. Isoelectric focusing was used to determine the isoelectropoint (pI) of the protein and was performed using a pH 3–10 isoelectric focusing (IEF) gel (Invitrogen, CA, USA). R27T in media samples was quantified using a rhIFN-β ELISA kit (IBL, Hamburg, Germany) according to the manufacturer’s instructions.

### Glycosylation Site Confirmation

LC/ESI MS/MS was used for site-specific glycosylation analysis of R27T. R27T was reduced and then digested with trypsin/Glu-C. Presence of carbohydrate specific fragment ions, such as *m/z* 204 and 366, in the product ion spectra, was analyzed from glycopeptide ions. For identification of the exact site of glycosylation, PNGase F was used to remove N-linked glycans from trypsin/Glu-C glycopeptides, and for conversion of Asn to Asp. Deglycosylated peptides were sequenced using LC/MS/MS.

### Monosaccharide and Sialic Acid Composition Analysis

A Dionex HPAEC (ThermoFisher Scientific, MA, USA) was used to analyze monosaccharide and sialic acid composition. AminoTrap Columns (ThermoFisher Scientific) and Carbopac PA10 analytical columns (ThermoFisher Scientific) were used at a flow rate of 1 mL/min at 30°C. Acidic sugars were analyzed by exposing the samples in 100 mM NaOH and 1 M NaOAc. All monosaccharides, including neutral and amino sugars, were analyzed by exposing the samples to 18 mM NaOH for 25 min. The waveform used for pulsed amperometric detection (PAD) was the Dionex default program for carbohydrates.

### Dynamic Light Scattering (DLS) and Atteuated Total Reflectance Fourier Transform Infrared (ATR-FTIR) Spectroscopy

Hydrodynamic size was investigated using a Zetasizer Nano ZS90 (Malvern Instruments, Baden-Württemberg, Germany). The temperature in the Zetasizer chamber was equilibrated to 10°C. Each sample was measured in a disposable sizing cuvette (Sarstedt, Germany). Hydrodynamic size and polydispersity index (PDI) were calculated from the auto-correlation function using Zetasizer software, version 6.32 (Malvern Instruments). ATR-FTIR spectra (4000–600 cm^–1^) were collected at 4 cm^–1^ resolution using a Nicolet 6700 spectrophotometer (ThermoFisher Scientific) with a golden gate accessory (diamond crystal). The α-helix, β-sheet, β-turn, and random coil contents of the proteins were estimated from the amide I region of ATR-FTIR spectra. Peaks of the amide I region were first treated by Fourier self-deconvolution and then curve-fitted using the Gauss and Lorentz formula with OMNIC Peak Resolve software (ThermoFisher Scientific). The area corresponding to each secondary structure was calculated accordingly and expressed as a percentage of the sum of areas. To measure kinetic thermostability, rhIFN-β 1a and R27T were incubated over 96 hr at 37°C and decay curves were generated for each protein. Detectable rhIFN-β was quantified by using the cytopathic effect (CPE) assay at each sampling time.

### *In vitro* Antiviral, Anti-proliferative and Immunomodulatory Activity

Antiviral activities were measured to determine the capacity of rhIFN-β to protect A549 cells against the CPE of a lytic virus over a range of rhIFN-β concentrations. World Health Organization natural rhIFN (NIBSC code: 00/572) was utilized as a standard. To measure antiviral activity, A549 cells were seeded in 96-well plates and serial dilutions of the proteins were added. Plates were incubated for 22 hr and encephalomyocarditis virus (EMCV, 1000 TCID 50/mL) was added. Following a further 22 hr incubation, cells were dyed with crystal violet, at room temperature for 1 hr, and then the dye was extracted with 2-methoxyethanol. Absorbance at 570 nm was then measured. For measurement of anti-proliferation effects, Daudi cells were seeded and serial dilutions of the proteins were added, followed by incubation for 48 hr. Cell proliferation assays were performed using an EZ-Cytox cell viability assay kit following the manufacturer’s protocol. Immunomodulatory effects of drugs were measured by analyzing the presentation of MHC Class I in A549 cells. A549 cells were seeded in 100 mm dishes and treated with a serial dilution of each protein, followed by incubation for 48 hr. After harvesting cells and adjusting each sample for equal cell density with FACS buffer, FACS was used to measure the expression of MHC class I peptides.

### Molecular Modeling of the R27T/IFNAR2 Complex

Molecular models of glycosylated rhIFN-β 1a were built from the crystal structure of wild-type rhIFN-β 1a (PDB ID: 1AU1) [21]. Mutation of arginine to threonine at the 27^th^ residue, and N-linked glycosylation of 1AU1 were performed using UCSF Chimera [22], [23] and GLYCAM [24]–[26]. Due to the high amino acid sequence identity (about 30%) between IFN-β 1a and IFN-α 2a, initial IFN-β 1a/IFNAR2 complex structures were generated using structural alignment with a model of IFN-α 2a/IFNAR2, whose structure was previously determined by NMR based docking methods (PDB ID: 2HYM) [27]. For modeling N-glycosylation, one of the major oligosaccharide structures (FA2G2S2, F: Core fucosylated, A2: biantennary with both GlcNAcs as b1–2 linked, G2: two galactose linked beta 1–4 to antenna, S2: two sialic acids linked to galactose) was chosen using a structure obtained from the hydrophilic interaction liquid chromatography (HILIC) profiles of R27T. The oligosaccharide was built using Carbohydrate Builder (Woods Group. 2005–2013, GLYCAM Web. Complex Carbohydrate Research Center, University of Georgia, Athens, GA., http://www.glycam.com) and was attached to the N-glycosylation site of the wild-type IFN-β 1a/IFNAR2 structure. The final structures were minimized using Amber force field (AMBER99SB) [28]. During minimization, Amber parameters were used for standard residues, and the Antechamber module was used to make parameters for non-standard residues. Steepest minimization was performed (using 100 steps) to relieve unfavorable clashes, followed by 100 steps of conjugate gradient minimization. Steepest descent and conjugate gradient minimization size was 0.02 Å. Molecular visualization was done using UCSF Chimera and PyMOL (The PyMOL Molecular Graphics System, Version 1.2r3pre, Schrödinger, CA, USA).

### *In vivo* Pharmacokinetic Analysis in Rats

Sprague Dawley rats were purchased from Orient Bio, Inc. (Gyeonggi-do, Korea), and maintained on a 12 hr light/dark cycle in a temperature- and humidity-controlled animal research facility. All rats were 8 weeks old, weighing 280–300 g at the beginning of each experiment. Each experiment included 9 groups (n = 3 per group). The femoral artery and vein were cannulated with a polyethylene tube (PE-50; Clay Adams, NJ, USA) under anesthetization. rhIFN-β substances (R27T, R27TΔGlyc and Rebif) were bolus-injected intravenously (via the femoral vein; IV), subcutaneously (at the abdomen; SC), or intramuscularly (at the leg; IM) at a dose of 1 MIU/kg to rats (total injection volume of approximately 0.3 mL). Approximately 120 µL of blood was collected via the femoral artery at 0 (to serve as a control), 1, 5, 15, 30, 60, 120, 240, 480, 720, and 1440 min after IV administration and at 0 (to serve as a control), 15, 30, 45, 60, 120, 180, 240, 360, 480, 720, and 1440 min after SC and IM administration. Approximately 0.3 mL of heparinized 0.9% NaCl-injectable solution (20 IU/mL) was used to flush each cannula immediately after blood sampling. Blood samples were centrifuged immediately, and a 50 µL aliquot of each plasma sample was stored in a −80°C freezer. All animals were humanely sacrificed at the end of experiments by euthanasia method: CO_2_ inhalation in a nonprecharged 4L chamber of a moderate fill rate. Standard non-compartmental analysis methods were used to calculate pharmacokinetic parameters (WinNonlin; standard version 3.1; Pharsight, CA, USA) [29], [30].

### Statistical Analysis

A p-value less than 0.05 was considered to be statistically significant. Statistical analysis was performed using Statistical Package for the Social Sciences (SPSS, IBM Corporation, NY, USA). Statistical significance was determined by analysis of variance (ANOVA) with Duncan’s multiple range test posteriori. All results were expressed as mean ± standard deviation, except for time to reach a C_max_ (T_max_), which was expressed as the median (ranges).

## Results

### Construction of the rhIFN-β Glycosylation Analogs, R27T

**Figure 1:**
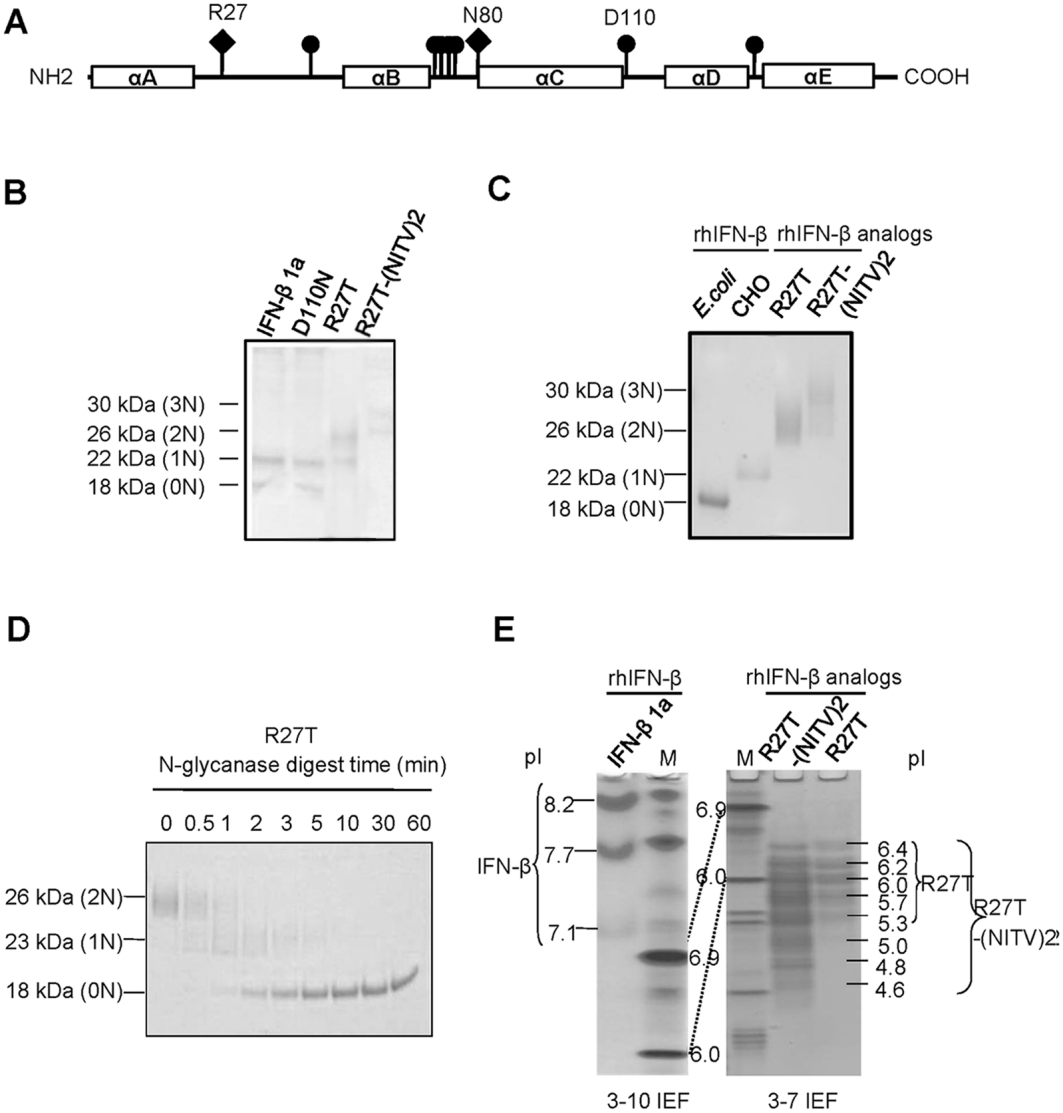
Construction and testing of rhIFN-β glycosylation analogs. (A) A schematic of the rhIFN-β protein. Boxes represented the locations of the five α-helices. Each vertical line represented a position with potential for an additional N-linked glycosylation site, as predicted by NetNGlyc. Introduced N-linked glycosylation consensus sequence sites were showed by diamonds(?). Purified samples were separated by (B) SDS-PAGE and (C) western blot analysis. (D) Analogs were subjected to N-glycanase digestion for the indicated times. (E) IEF analysis was performed over a pH range of 3–10.

To best maintain the structural and functional properties of the protein, additional N- glycosylation sites were not created in the five helical regions, consisting of amino acid residues A(2–22), B(51–71), C(80–107), D(112–136) and E(139–162). A number of sites including Arg27, Asp39, Gln72, Asp73, Ser74, Ser75, Asp110 and Glu137 were then screened for the additional introduction of a consensus N-glycosylation sequence (Asn-X-Ser/Thr, where X is any amino acid except Pro). Each site was analyzed using NetNGlyc software on the Center for Biological Sequence Analysis website (Technical University of Denmark) (Table S1). Based on our analysis, we selected the most flexible overhand AB loop and the highest mobility CD loop to minimize the structural modification, remaining at Arg27, Asp39 and Asp110 (Figure 1A). A substitution at Arg27 of rhIFN-β 1a was chosen as the most promising site, as it had the highest substitution potential score in NetNGlyc, whereas Asp110 was chosen as a negative control. The substitutions made were Arg27 and Asp110 with Thr and Asn, respectively. In addition, a hyper-glycosylated rhIFN-β model was created by extension of the sequence “ANITVNITV, termed (NITV)_2_” into the C-terminus. Both R27T and R27T-(NITV)_2_ showed a clear increase in molecular weight (by 26 and 30 kDa, respectively) in western blots (Figure 1B). No change in molecular weight was observed for the D110N analog, compared with native rhIFN-β, at 22 kDa. This was confirmed by SDS-PAGE of rhIFN-β after purification (Figure 1C). Treatment with PNGase, which catalyzes the release of N-linked oligosaccharides, resulted in the appearance of three bands of 26, 22 and 18 kDa in the R27T sample (Figure 1D). The upper and middle bands were doubly or singly glycosylated respectively, whereas the lower band contained only the peptide. Thus the molecular weight increment was attributed to additional glycosylation. Negative charge was increased in R27T and R27T-(NITV)_2_, which had pI values of 5.3–6.4 and 4.6–6.4 respectively, compared to native rhIFN-β, with a pI of 7–8 (Figure 1E). Notably, R27T, which had one additional glycan at the 25^th^ amino acid, was selected as the lead protein because the amino acid change for the additional glycosylation site was minimal. This could be advantageous, as it could result in lower immunogenicity than that for R27T-(NITV)_2_.

### Glycosylation Site Confirmation of R27T Analogs with Additional Glycosylation

**Figure 2:**
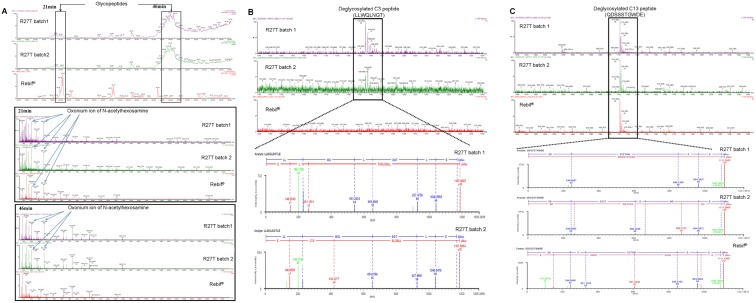
LC/ESI MS/MS of trypsin/Glu-C digests of R27T and Rebif. (A) Extraction ion chromatogram at m/z 204 and 366 for product ion spectra at 21 min and 46 min, respectively. Amino acid sequencing of deglycosylated C3 and C13 peptides by LC/ESI MS/MS. (B) Deglycosylated C3 peptide MS spectrum and fragmentation information for R27T (Lot: 12104DS01 and 12103DS01) and Rebif. (C) MS spectrum of the deglycosylated C13 peptide and fragmentation information for R27T (Lot: 12104DS01 and 12103DS01) and Rebif.

Specific glycosylation sites were conclusively confirmed by determination of the carbohydrate attachment site on the polypeptide backbone of the protein. Glycopeptides were identified by performing quadrupole-time of flight (Q-TOF) MS on trypsin/Glu-C digests of R27T and the control Rebif, in which the C3 and C13 peptide fragment had potential glycosylation sites at Asn25 and Asn80 in R27T, and Asn80 in Rebif (Figure S1A and S1B). Glycopeptide ions were extracted and confirmed by examining for the presence of oxonium ions, such as *m/z* 204 (HexNAc) and 366 (HexHexNAc), with retention times of 21 min and 46 min, respectively, on the extracted ion chromatogram (Figure 2A). The presence of oxonium ions was again determined in the product ion spectrum at 21 min and 46 min (Figure 2A). The identification of N-glycosylation sites by MS depends on the specific deamidation of asparagines to aspartic acid within the consensus sequence NX(S/T) upon cleavage of the glycan moiety by PNGase F. The deglycosylated peptide was sequenced by LC/MS/MS (Figure 2B and 2C). Glycosylation sites of R27T were identified at Asn25 and Asn80 in R27T.

### Monosaccharide and Sialic Acid Composition Analysis

We performed glycosylation analysis to compare the relative proportion of monosaccharide content, mono-, di-, tri- and tetra-antennary structures with those of the reference, Rebif. Individual sugar residues were identified and quantitated as moles of monosaccharide per moles of protein. The monosaccharide contents in 1 mol of R27T and Rebif were shown in Table 1. The contents of each monosaccharide and sialic acid per moles of protein were increased with additional glycosylation, compared to Rebif. All carbohydrate moieties consisted of fucose, N-acetylglucosamine, galactose, mannose and sialic acid without N-acetylgalactosamine. Therefore, they were N-linked complex-type sugar chains.

**Table 1:** Sugar composition analysis of R27T and Rebif (mol/mol protein).

	Fucose^a^	N-acetyl-glucosamine^b^	N-acetyl-galactosamine^b^	Galactose^a^	Mannose^a^	Sialic acid^c^
R27T	2.4	12.3	N.D.	7.7	7.1	3.2
Rebif	1.1	4.7	N.D.	3.0	3.5	1.2

a 2 M TFA for neutral sugars at 100°C at 4 hr.

b 6 N HCl for amino sugars at 100°C at 4 hr.

c 0.1 N HCl for sialic acids(NANA+NGNA) at 80°C at 1 hr.

### Biophysical Analysis of Protein Stability by DSC, DLS and ATR-FTIR

**Figure 3:**
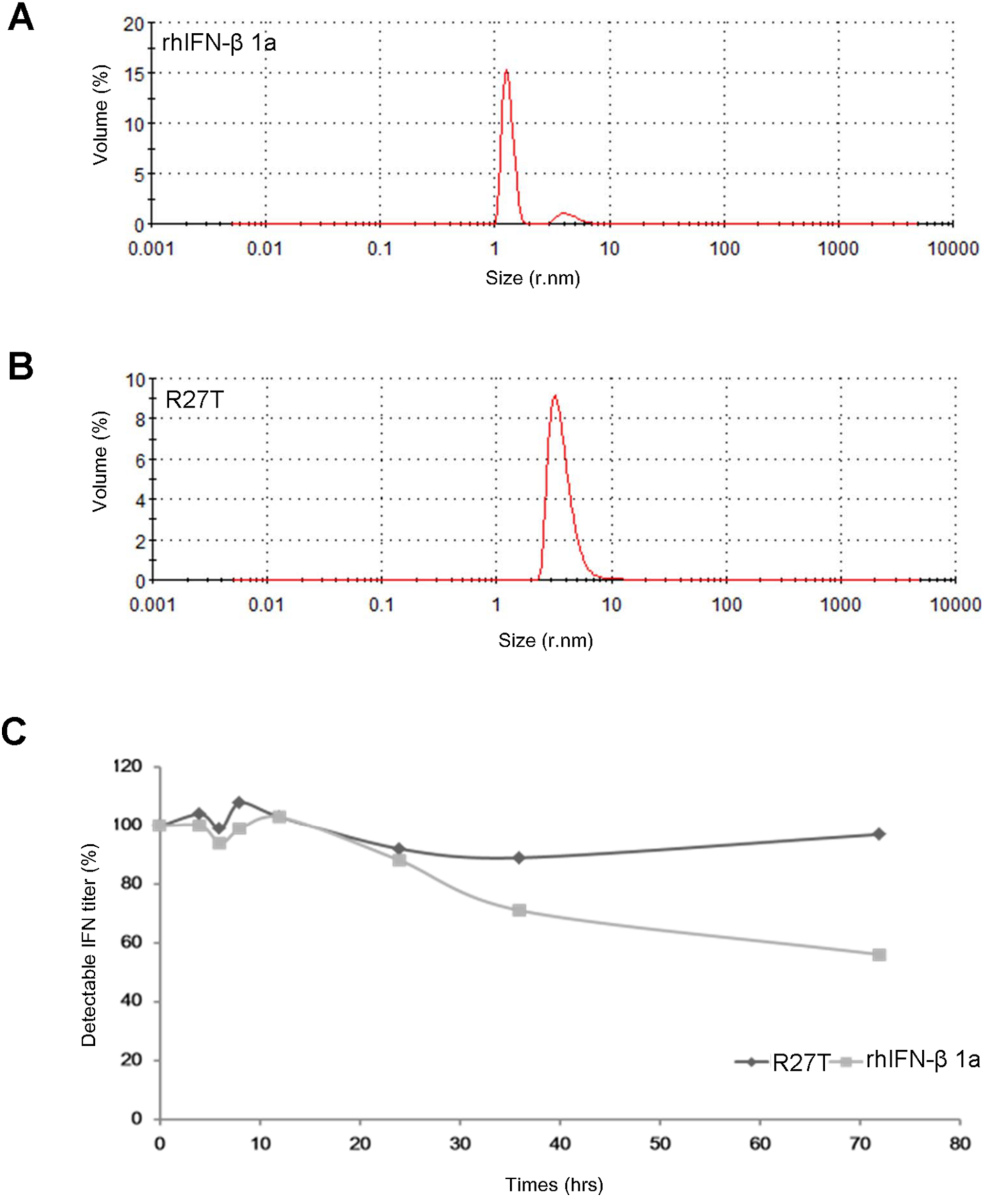
Size distribution of rhIFN-β 1a and R27T. The analysis of (A) rhIFN-β 1a and (B) R27T was performed at a scattering angle of 90° and distributed sizes, PDI, and Zeta averages were shown. (r.nm: radius in nanometers). (C) rhIFN-βs were quantified by CPE at each time up to 72 hr.

DSC thermograms of rhIFN-β 1a and R27T in 20 mM acetate buffer pH 4.2 were evaluated to obtain thermal unfolding events (Figure S2). Unfolding transition temperature (*T_m_*), calorimetric enthalpy (ΔH) and van’t Hoff enthalpy (ΔH_v_) of rhIFN-β 1a were 61.90°C, 39.88 kcal/mol and 106.3 kcal/mol, respectively. In addition, *T_m_,* ΔH and ΔH_v_ of R27T were 59.07°C, 36.87 kcal/mol and 103.6 kcal/mol, respectively. Even though R27T exhibited lower *T_m_* than rhIFN-β 1a while having almost similar ΔH and ΔH_v,_ it did not exhibit any visible particles after withdrawn from the DSC scan (data not provided). DLS was used to observe hydrodynamic size of rhIFN-β 1a and R27T and existence of aggregates in the aqueous environment as well. DLS measurements of the proteins were provided in Figure 3. rhIFN-β 1a gave two volume distribution peaks at around 2.700 nm and 9.825 nm with 98.9% and 1.3% volume ratio, respectively. However, R27T had only single size distribution peak at around 3.722 nm by volume distribution.

Polydispersity index (PDI) is dimensionless and scaled from 0 to 1 where values less than 0.05 are rarely seen other than with highly monodisperse standards. However, values higher than 0.7 suggest that the sample has a very polydispersed distribution and is not suitable for the DLS measurement. PDI values of rhIFN-β 1a and R27T were 0.607 and 0.306, respectively. Since rhIFN-β 1a had higher PDI value than R27T, the protein was more polydispersed and might not be stable in the aqueous environment compared to R27T. Therefore, the result may indicate that rhIFN-β 1a possess instability issue in the aqueous solution compared to R27T. In order to investigate secondary structural stability of the proteins, ATR FT-IR was selected to analyze the amide group I (1700–1600 cm^−1^) in proteins. The amide group I region of ATR-FTIR spectra was separated into nine peaks with Fourier self-deconvoluted spectra of rhIFN-β 1a and R27T. Each ratio of composite area represented the corresponding percentage of each structure from peak 1 to 9; peak #1, 2, 6 and 7 (1692 cm^−1^, 1676 cm^−1^, 1636 cm^−1^ and 1623 cm^−1^, respectively; β-sheet), peak #3 (1665 cm^−1^; reverse turn), peak #4 (1655 cm^−1^; α-helix), peak #5 (1646 cm^−1^; random coil), peak #8 (1615 cm^−1^; side chain vibration) and peak #9 (1598 cm^−1^; β-turn) were resolved accordingly. After resolving the peaks, relative percentage of the contents was calculated. Table 2 showed the relative ratio of α-helix, β-sheet, β-turn and random coil of rhIFN-β 1a and R27T. R27T contained more α-helix and less β-sheet than rhIFN-β 1a. It was already known from the crystal structure of rhIFN-β 1a that there was no β-sheet secondary structure. However, recent spectroscopy studies give that increasing intermolecular β-sheet structure is a common feature of protein aggregation although interferon consists of only α-helix [31]–[33]. Since β-sheet contents of rhIFN-β 1a were about 5.69% higher than R27T, it might suggests higher potential of protein aggregations [32].

**Table 2:** Secondary structure ratios derived from the ATR-FTIR spectra of rhIFN-β 1a and R27T in solution.

Samples	Amide I region in ATR FTIR spectra			
	α-helix (%)	β-sheet (%)	β-Turn (%)	Random Coil (%)
rhIFN-β1a	17.3	37.0	24.5	21.2
R27T	29.8	31.3	25.6	13.3

To confirm the actual effect of stability issue, the aggregation kinetics was shown in figure 3C. CPE assay was used to detect bioactivity of R27T and rhIFN-β 1a in accelerated condition 37°C up to for 96 hr. The bioactivity with aggregation of R27T compared with rhIFN-β 1a had a less decrease within the first 96 hr. The calculated half-life for rhIFN-β 1a was 96 hr in contrast to 503 hr for R27T.

### Maintenance of *In vitro* Activity

**Figure 4:**
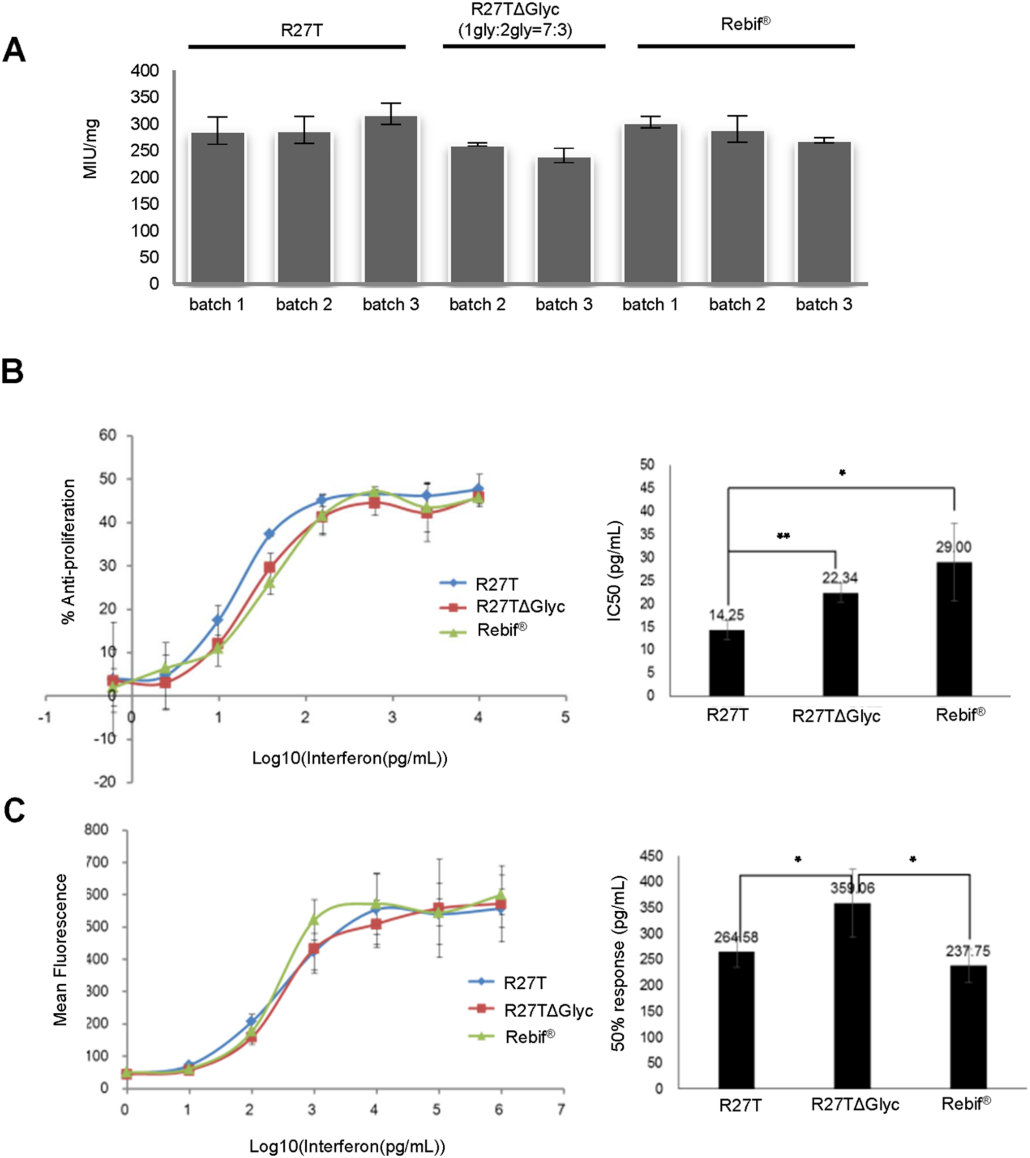
*In vitro* activity. (A) Anti-viral, (B) anti-proliferative and (C) immunomodulatory activities of R27T (?), R27TΔGlyc (▪) and Rebif (▴).

R27T and Rebif was analyzed for *in vitro* anti-viral activity together with an R27TΔGlyc. Values of 299×10^6^, 252×10^6^ and 288×10^6^ IU/mg were obtained from the average activity of the three lots of R27T, two lots of R27TΔGlyc and Rebif, respectively (Figure 4A). No decrease in antiviral activity was observed with additional glycosylation. We also examined the relative potencies of R27T, R27TΔGlyc, and Rebif in anti-proliferative and immunomodulation assays. Similar activities were observed in these assays. In the antiproliferative assay, IC50s of 14, 22 and 29 pg/mL were obtained for R27T, R27TΔGlyc and Rebif respectively (Figure 4B). In a FACS assay, which measured IFN-inducible expression of MHC class I on the surface of A549 cells, 50% responses were observed at approximately 265, 359 and 238 pg/mL, respectively (Figure 4C).

### Molecular Modeling of the R27T/IFNAR2 Complex

**Figure 5:**
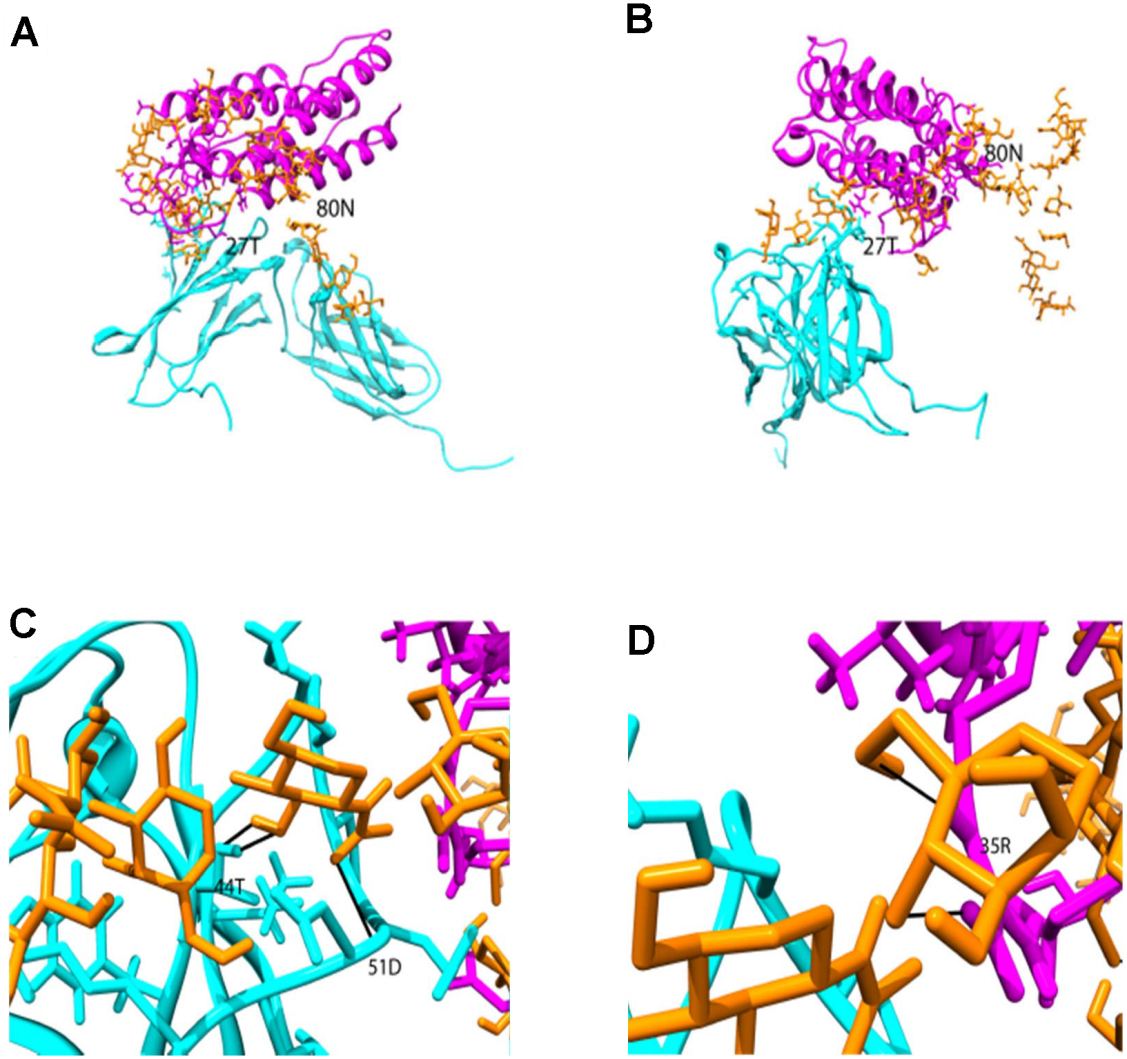
Model of the N-glycosylated R27T/IFNAR2 complex. (A) front view and (B) side view. The complex structure oligosaccharides, R27T, and IFNAR2 were shown as orange, magenta, and cyan color, respectively. New hydrogen bonds between the oligosaccharide and complex were presented as red bold line in C (with IFNAR2) and D (R27T). Also, 27T and 80N in R27T protein were colored by yellow (A,B) and hydrogen-bonded residues in R27T and IFNAR2 were colored by green (C,D).

An R27T structure was generated *in silico*, based on the crystal structure of human IFN-β 1a. The R27T/IFNAR2 docking structure was shown in Figure 5A and 5B. N-glycosylation was predicted to readily occur at the 80^th^ amino acid, regardless of oligosaccharide structure or the distance (solvent accessible surface area 76.7Å by Naccess V2.1.1.1) at which glycosylation could have no effect on the R27T/IFNAR2 complex due to far distance between oligosaccharide and receptor [34]. By contrast, glycosylation at the 25^th^ amino acid was more difficult to model *in silico* than at the 80^th^ residue, as the 25^th^ amino acid side chain was oriented towards the inside of the protein. However, “wet” experiments, including SDS-PAGE, confirmed that it was possible to glycosylate the 25^th^ amino acid, in a similar manner to that of the 80^th^ amino acid (Figure 1B and 1C). A structure for R27T with the 25^th^ residue glycosylated was obtained from the *in silico* data. This showed that glycosylation of R27T at the 25^th^ amino acid could stabilize the interaction between R27T and IFNAR2. In particular, new hydrogen bonds were formed between Thr44 and Asp51 of IFNAR2 and the oligosaccharide moiety of R27T, thereby increasing R27T/IFNAR2 complex stability (Figure 5C). Similarly, hydrogen bonding between Arg35 of R27T and the oligosaccharide moiety contributed towards the overall stability of the helical conformations within R27T (Figure 5D).

### *In vivo* Pharmacokinetic Studies in Rats

**Figure 6:**
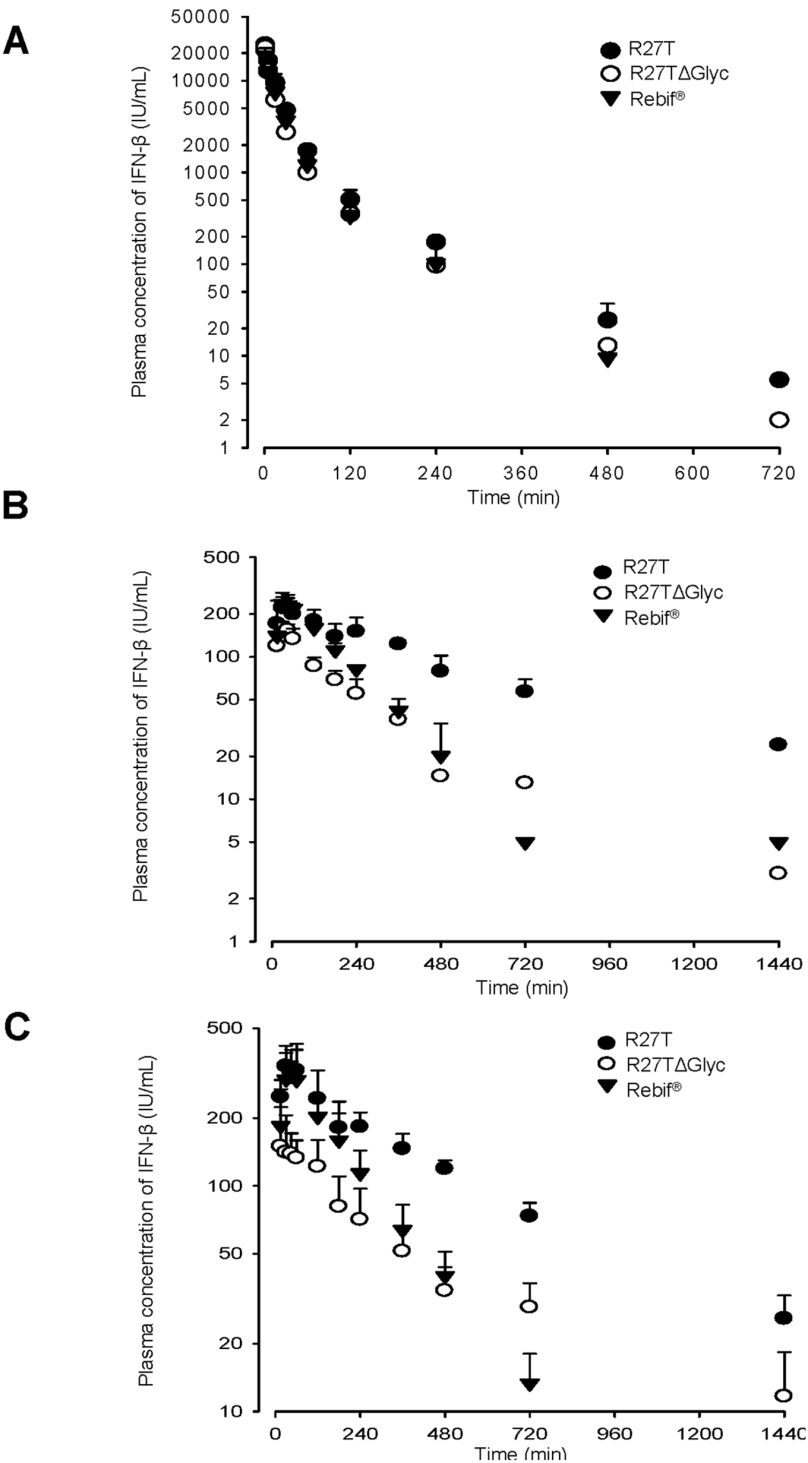
Mean arterial plasma concentration–time profiles for rhIFN-βs. (A) IV, (B) SC and (C) IM administration of R27T, R27TΔGlyc and Rebif at a dose of 1 MIU/kg in rats. Vertical bars indicated standard deviations.

Plasma concentration-time profiles for the rhIFN-β variants were obtained in rats after IV, SC and IM administration of R27T, R27TΔGlyc and Rebif at a dose of 1 MIU/kg (Figure 6). Relevant pharmacokinetic parameters were listed in Table 3. After IV administration of the three rhIFN-βs formulations, bi-exponential declines in plasma IFN concentration were observed. The area under curve (AUC) values for R27T were significantly higher than those of R27TΔGlyc and Rebif by 1.47 and 1.36 fold, respectively. Thus, the total body clearance (CL) values were significantly lower in R27T than in the other rhIFN-β formulations. Moreover, the terminal half-life (t_1/2_) values of R27T tended to be higher than those of the other rhIFN-β formulations by 1.39 and 1.44 fold, respectively. The apparent volume of distribution at steady state (V_ss_) values were comparable among the three rhIFN-β formulations. After SC administration, the AUC and extent of absolute bioavailability (F) values of R27T were significantly higher than those of R27TΔGlyc by 2.95 and 2.01 fold, respectively, and those of Rebif by 2.33 and 1.72 fold, respectively. Moreover, the t_1/2_ values of R27T tended to be higher than those of R27TΔGlyc and Rebif by 1.71 and 2.35 fold, respectively. The peak plasma concentration (C_max_) and T_max_ values were comparable among the three rhIFN-β formulations. After IM administration, the AUC and F values of R27T were significantly higher than those of R27TΔGlyc by 2.36 and 1.58 fold, respectively, and those of Rebif by 2.24 and 1.65 fold, respectively. The t_1/2_ values of R27T and R27TΔGlyc were significantly higher than those of Rebif by 2.84 and 3.56 fold, respectively. The C_max_ values of R27TΔGlyc were significantly lower than those of R27T and Rebif, and the T_max_ values were comparable among the three rhIFN-β formulations. The t_1/2_ values after SC and IM administration were much higher than those after IV administration, indicating that each substance is subject to flip-flop kinetics.

**Table 3:** Pharmacokinetic parameters for IFN-β after IV, SC, and IM administration of R27T, R27TΔGlyc, and Rebif at a dose of 1 MIU/kg to rats (n = 3).

Parameters	R27T	R27TΔGlyc	Rebif
IV			
AUC (×10^4^ IU•min/mL)	54.8±7.4*	37.2±2.1	40.3±2.4
t_1/2_ (min)	88.0±4.4	63.3±11.5	61.3±21.6
CL (mL/min/kg)	1.9±0.2*	2.7±0.1	2.5±0.2
V_ss_ (mL/kg)	90.1±8.7	110±17.1	107±36.2
SC			
AUC (×10^4^ IU•min/mL)	12.2±1.5*	4.1±1.3	5.2±0.5
t_1/2_ (min)	423±154	247±121	180±69.3
C_max_ (IU/mL)	235±54.0	161±14.9	246±42.0
T_max_ (min)	30 (15–45)	30 (30)	45 (30–45)
F (%)	22.3	11.1	13.0
IM			
AUC (×10^4^ IU•min/mL)	16.3±2.0*	6.9±1.3	7.3±2.1
t_1/2_ (min)	426±44.7	534±211	150±11.8*
C_max_ (IU/mL)	374±59.7	172±35.1*	314±98.4
T_max_ (min)	45 (30–60)	15 (15–120)	45 (30–60)
F (%)	29.7	18.8	18.0

*Significantly different to the other groups (p<0.05).

## Discussion

We have constructed a biobetter version of rhIFN-β 1a, termed R27T, as evidenced the results of structure-function studies of rhIFN-β 1a and the type I IFN-β receptor. R27T contained a substitution of Thr for Arg at position 27^th^ of rhIFN-β 1a, which resulted in additional glycosylation at the 25^th^ position. Glycoengineering was successfully performed, as demonstrated by the bioassay, MS spectrometry and *in* *vitro* data. Notably, the *in* *vitro* assays, including measures of antiviral, anti-proliferation and immunomodulation, showed that additional glycosylation at the 25^th^ amino acid (as a result of the R27T mutation) had no effect on ligand-receptor binding. However, these results were different to the results of Runkel et al [35], [36]. Using alanine-scanning mutagenesis, they showed that the 27^th^ amino acid, arginine, was a solvent exposed residue important for antiviral and reporter gene activity. Although alanine-scanning mutagenesis is a widely used technique to determine the functional role of each amino acid, mutagenesis of the Arg27, which has a polar residue group, to alanine, which has nonpolar, aliphatic residue group, could cause different effects than mutation into threonine, which has a polar residue group. In addition, glycosylation at the 25^th^ amino acid could cause conformational changes without influencing receptor binding.

Glycosylation could also affect molecular stability. Stability issues, such as aggregation, have been one of the biggest challenges for the production of therapeutic proteins. Moreover, these issues occur at very different stages in the development processes, including drug production, purification, storage and delivery. To address these issues, the behavior of rhIFN-β 1a and R27T in solution was investigated using biophysical methods. Conformational stability (*T_m_* value) of R27T by glycoengineering compared to rhIFN-β 1a was observed by DSC. *T_m_* of R27T was less than that of rhIFN-β 1a. Although glycosylation generally enhance the conformational stability, the position of glycosylation on protein is known to be a key factor to modulate the stability [37]. Thus, additional glycosylation at Asn25 on R27T seemed to have less effect on conformational stability. However, DLS and ATR-FTIR data suggested that rhIFN-β 1a was more susceptible to biophysical stability, such as aggregation, than R27T. In particular, ATR-FTIR indicated that a significant amount of α-helical structure had been retained, whereas intermolecular β-sheet content (which reflects aggregation) was decreased by the additional glycosylation (at the 25^th^ amino acid). These results were consistent with the DLS data since rhIFN-β 1a was more polydispersed than R27T. Furthermore, these results were also confirmed by the aggregation kinetic profiles, which demonstrated the beneficial effect of additional glycosylation on stability during accelerated storage conditions. This effect could be caused by enhanced biophysical stability of R27T by glycoengineering. In addition, R27T productivity was approximately 3–6 times higher than that of rhIFN-β 1a under low temperature conditions of 30°C–34°C for 6–14 days without additional manipulations like low temperature adapted cells, using cytopore, intermediate temperature shift and perfusion for production (Table 4). It was likely that this was a consequence of the lower aggregation resulting from the additional glycosylation, since it was well known that the low productivity of rhIFN-β 1a was caused by molecular aggregation.

**Table 4:** Comparative rhIFN- β 1a productivity in CHO cells; rhIFN- β productivity in CHO cells was compared.

Culture condition	Temperature(°C)	Additives	Cultureperiod(day)	Max celldensity(cells/mL)	SpecificProductivityQp (β-IFNunits/cell/day)	Total volumetric production(×10∧6 units/ml)		End cultureviability (%)	Reference
						(×10∧6 units/mL,Non-denatured)	(×10∧6 units/mL,denatured)		
Batch (Spinner Flask)	34		6	2.00E+06		10			
Batch (Bioreactor 7.5 L)	34		6	4.00E+06		16			In our study
Batch (Bioreactor 30 L)	34		6	4.00E+06		9			
Batch (Spinner Flask)	37		7	3.00E+06		1	3.7		
Batch, Microcarrier(Cytopore 1 1 mg/ml)	37		7	1.50E+06		5	14		
Batch, Microcarrier(Cytopore 2 1 mg/ml)	37		7	1.50E+06		5	8		Maureen Spearman et al, Biotech. Prog. 2005, 21,31–39
Batch (Bioreactor 2 L)	37		7	3.80E+06		1.5	7.5		
Batch, Microcarrier(Cytopore 1 1 mg/ml)	37		7	3.00E+06		5.8	10		
Batch (Bioreactor 2 L)	37		7	4.20E+06*	0.95	1.3	14		
Batch (Bioreactor 2 L)	37	2% Glycerol	7	3.20E+06*	0.85	6.7	9.6		
Batch (Bioreactor 2 L)	37	1% DMSO	7	2.00E+06*	2	2.5	12.8	85%	J. Rodriguez et al, Biotechnol. Prog. 2005,21,22–30
Batch (Bioreactor 2 L)	37	1 mM NaBu	5	1.30E+06*	3.5	1.5	8.9		
Batch (Spinner Flask)	37		8	3.50E+06		0.5	1.5		
Batch (Spinner Flask)	30		14	7.50E+05		3.3	3.6	>90%	
Batch (Spinner Flask)	37 → 30 shift		12	1.80E+06		4.2	9		
Batch (Bioreactor 2 L)	37		7	3.20E+06		0.4	3		Maureen Spearman et al, Cell Technology for Cell Products,2007, 71∼85
Batch (Bioreactor 2 L)	37	1 mM NaBu	5	1.50E+06		2	7.5		
Batch (Bioreactor 2 L)	37	40 mM NaCl	7	2.30E+06		2.1	5.5		
Batch, Microcarrier(Cytopore 1 1 mg/mL)	37		7	2.50E+06		2.2	4		
Batch (Spinner Flask)	37		6	3.45E+06		0.96	1.7		
Batch (Spinner Flask)	37 → 30 shift		6	1.80E+06		4.4	5.6	>90%	
Batch (Spinner Flask)	30		10	6.90E+05		1.5	2.35		
Batch (Spinner Flask)	37	2% Glycerol	6	2.05E+06		1.7	4.5		
Batch (Spinner Flask)**	32		10	1.6E+05		2.2		93%	
Batch (Spinner Flask)**	37 → 32 shift		10	6.9E+05		2.4		98%	
Batch (Spinner Flask)**	37		6	4.1E+06	0.341	4.24			
Batch, Microcarrier (Cytopore 2 1 mg/ml)**	37		6	2.5E+06	0.954	5.19			
Batch(Spinner Flask)**	32		12	1.7E+05	0.784	1.32			Kelvin Sunley et al., Biotechnol. Prog. 2008, 24,898–906
Batch, Microcarrier (Cytopore 2 1 mg/ml)**	32		12	8.2E+05	0.847	3.27			
Batch (Spinner Flask)***	37		10	1.1E+06		5.7		93%	
Batch (Spinner Flask)***	37 → 32 shift		10	2.5E+06		7.1		88%	
Batch (Spinner Flask)***	32		12	2.5E+05	0.233	0.73			
Batch, Microcarrier (Cytopore 2 1 mg/ml)***	32		12	5.0E+06	0.92	10.1			
Batch (Bioreactor 2 L)	37		0–3	1.39E+06	0.75	0.9	1.5		
Batch (Bioreactor 2 L)	32		3–8	2.55E+06	1.2	12.4	17.4		J. Rodriguez et al, Journal of Biotechnology 2010,150,120
Perfusion(Bioreactor 2L)	32		8–16	3.70E+06	2.6	9.3	9.9	>95%	

The cultures of R27T were established in a shake flask and 7.5 L (working volume: 5 L), or 30 L (working volume: 20 L) bioreactors. For ELISA, R27T was obtained from bioreactors on day 7, or from shake flasks on day 6 of culture.

Atmosphere: 10% CO2.

*Final Cell Yield(cells/mL).

**Low Temperature Non-adopted.

***Low Temperature adopted.

To investigate the effect of glycosylation at the 25^th^ amino acid on stability, a computational simulation of R27T/IFNAR2 docking was performed. Intrinsic stability was improved by glycosylation at the 25^th^ amino acid, which enabled hydrogen bonding between residue 35R on R27T and the oligosaccharide. Structural simulations of R27T/IFNAR2 suggested that the oligosaccharide could also interact with IFNAR2, resulting in the stabilization of ligand-receptor binding interactions. These results imply that glycosylation may be important for fine-tuning glycoprotein bioactivity although X-ray crystallography experiments may be required to obtain a more detailed structure [38].

Glycans play a very prominent role in determining therapeutic efficacy, including the *in vivo* half-life. We therefore compared the pharmacokinetic properties of R27T, R27TΔGlyc and Rebif. After IV administration, the pharmacokinetic parameters (CL, V_ss_, and t_1/2_) of Rebif (1 MIU/kg) were consistent with those obtained in a previous study on the pharmacokinetics of native rhIFN-β, administered at a dose of 21 MIU/kg [39]. The AUC and CL values of R27T were significantly higher and lower, respectively, than those of Rebif and R27TΔGlyc. Although little information on the elimination route of rhIFN-β 1a is currently available, the reduced CL is likely due to the fact that R27T is a hyper-glycosylated form of rhIFN-β. The reduced CL may be attributed to the increased molecular weight and/or sialylation status. It has also been reported that the CL of hyper-glycosylated erythropoietin is significantly lower than that of native erythropoietin [40], [41]. In this regard, sialylation status could be a critical pharmacokinetic parameter [42], [43]. In fact, the terminal monosaccharide of the N-linked complex glycan of R27T was occupied by a sialic acid moiety, which could affect absorption, serum half-life and clearance as well as various other physicochemical properties. Moreover, after SC and IM administration, the AUC and F values of R27T were significantly higher than those of Rebif and R27TΔGlyc. This result could be attributed to the lower CL and/or higher stability of R27T compared to that of Rebif. However, the exact reasons for the increased SC and IM absorption, and reduced elimination of R27T were unclear; thus, further investigation is required. Taken together, after IV, SC, and IM dosing in rats, R27T achieved a higher and more prolonged systemic rhIFN-β 1a exposure than Rebif or R27TΔGlyc. This demonstrates the potential of R27T as a long-acting IFN analog.

## Supporting Information

Figure S1 **Amino acid sequence of Rebif and R27T.** Shadow on amino acid showed predicted glycopeptides and circles showed potential glycosylation sites of (A) Rebif and (B) R27T.
(TIF)

Figure S2 **DSC thermograms of rhIFN-β 1a and R27T.** Measured *T_m_* values of (A) rhIFN-β 1a and (B) R27T was displayed above the peaks. Graphs also showed curve fitting of the DSC thermograms for rhIFN-β 1a and R27T, which was performed using a non-two-state model.
(TIF)

Table S1 **Glycosylation site prediction.**
(DOCX)

Materials & Methods S1 (DOCX)

## References

[1] Noseworthy JH, Lucchinetti C, Rodriguez M, Weinshenker BG (2000) Multiple sclerosis. N Engl J Med 343: 938–952. 11006371 10.1056/NEJM200009283431307

[2] Fitzner D, Simons M (2010) Chronic progressive multiple sclerosis - pathogenesis of neurodegeneration and therapeutic strategies. Curr Neuropharmacol 8: 305–315. 21358979 10.2174/157015910792246218 PMC3001222

[3] Amedei A, Prisco D, D’Elios MM (2012) Multiple sclerosis: the role of cytokines in pathogenesis and in therapies. Int J Mol Sci 13: 13438–13460. 23202961 10.3390/ijms131013438 PMC3497335

[4] de Seze J, Borgel F, Brudon F (2012) Patient perceptions of multiple sclerosis and its treatment. Patient Prefer Adherence 6: 263–273. 22536062 10.2147/PPA.S27038 PMC3333819

[5] Castro-Borrero W, Graves D, Frohman TC, Flores AB, Hardeman P, et al (2012) Current and emerging therapies in multiple sclerosis: a systematic review. Ther Adv Neurol Disord 5: 205–220. 22783370 10.1177/1756285612450936 PMC3388530

[6] Gasperini C, Ruggieri S (2011) Emerging oral drugs for relapsing-remitting multiple sclerosis. Expert Opin Emerg Drugs 16: 697–712. 22148963 10.1517/14728214.2011.642861

[7] Nicholas R, Giannetti P, Alsanousi A, Friede T, Muraro PA (2011) Development of oral immunomodulatory agents in the management of multiple sclerosis. Drug Des Devel Ther 5: 255–274. 10.2147/DDDT.S10498 PMC3100222 21625416

[8] Harris JM, Martin NE, Modi M (2001) Pegylation: a novel process for modifying pharmacokinetics. Clin Pharmacokinet 40: 539–551. 11510630 10.2165/00003088-200140070-00005

[9] Watt GM, Lund J, Levens M, Kolli VS, Jefferis R, et al (2003) Site-specific glycosylation of an aglycosylated human IgG1-Fc antibody protein generates neoglycoproteins with enhanced function. Chem Biol 10: 807–814. 14522051 10.1016/j.chembiol.2003.08.006

[10] Katre NV (1993) The Conjugation of Proteins with Polyethylene-Glycol and Other Polymers - Altering Properties of Proteins to Enhance Their Therapeutic Potential. Advanced Drug Delivery Reviews 10: 91–114.

[11] Kieseier BC, Calabresi PA (2012) PEGylation of interferon-beta-1a: a promising strategy in multiple sclerosis. CNS Drugs 26: 205–214. 22201341 10.2165/11596970-000000000-00000

[12] Karpusas M, Whitty A, Runkel L, Hochman P (1998) The structure of human interferon-beta: implications for activity. Cell Mol Life Sci 54: 1203–1216. 9849615 10.1007/s000180050248 PMC11147404

[13] Runkel L, Meier W, Pepinsky RB, Karpusas M, Whitty A, et al (1998) Structural and functional differences between glycosylated and non-glycosylated forms of human interferon-beta (IFN-beta). Pharm Res 15: 641–649. 9587963 10.1023/a:1011974512425

[14] Derynck R, Remaut E, Saman E, Stanssens P, De Clercq E, et al (1980) Expression of human fibroblast interferon gene in Escherichia coli. Nature 287: 193–197. 6159534 10.1038/287193a0

[15] Mark DF, Lu SD, Creasey AA, Yamamoto R, Lin LS (1984) Site-specific mutagenesis of the human fibroblast interferon gene. Proc Natl Acad Sci U S A 81: 5662–5666. 6091102 10.1073/pnas.81.18.5662 PMC391770

[16] Kagawa Y, Takasaki S, Utsumi J, Hosoi K, Shimizu H, et al (1988) Comparative study of the asparagine-linked sugar chains of natural human interferon-beta 1 and recombinant human interferon-beta 1 produced by three different mammalian cells. J Biol Chem 263: 17508–17515. 3182859

[17] Sola RJ, Griebenow K (2009) Effects of glycosylation on the stability of protein pharmaceuticals. J Pharm Sci 98: 1223–1245. 18661536 10.1002/jps.21504 PMC2649977

[18] Wang W (2005) Protein aggregation and its inhibition in biopharmaceutics. Int J Pharm 289: 1–30. 15652195 10.1016/j.ijpharm.2004.11.014

[19] Rodriguez J, Spearman M, Tharmalingam T, Sunley K, Lodewyks C, et al (2010) High productivity of human recombinant beta-interferon from a low-temperature perfusion culture. J Biotechnol 150: 509–518. 20933553 10.1016/j.jbiotec.2010.09.959

[20] Rodriguez J, Spearman M, Huzel N, Butler M (2005) Enhanced production of monomeric interferon-beta by CHO cells through the control of culture conditions. Biotechnol Prog 21: 22–30. 15903237 10.1021/bp049807b

[21] Karpusas M, Nolte M, Benton CB, Meier W, Lipscomb WN, et al (1997) The crystal structure of human interferon beta at 2.2-A resolution. Proc Natl Acad Sci U S A 94: 11813–11818. 9342320 10.1073/pnas.94.22.11813 PMC23607

[22] Pettersen EF, Goddard TD, Huang CC, Couch GS, Greenblatt DM, et al (2004) UCSF Chimera–a visualization system for exploratory research and analysis. J Comput Chem 25: 1605–1612. 15264254 10.1002/jcc.20084

[23] Sanner MF, Olson AJ, Spehner JC (1996) Reduced surface: an efficient way to compute molecular surfaces. Biopolymers 38: 305–320. 8906967 10.1002/(SICI)1097-0282(199603)38:3%3C305::AID-BIP4%3E3.0.CO;2-Y

[24] Kirschner KN, Woods RJ (2001) Quantum mechanical study of the nonbonded forces in water-methanol complexes. J Phys Chem A 105: 4150–4155. 16518456 10.1021/jp004413y PMC1388248

[25] Basma M, Sundara S, Calgan D, Vernali T, Woods RJ (2001) Solvated ensemble averaging in the calculation of partial atomic charges. J Comput Chem 22: 1125–1137. 17882310 10.1002/jcc.1072 PMC1986576

[26] Kirschner KN, Woods RJ (2001) Solvent interactions determine carbohydrate conformation. Proc Natl Acad Sci U S A 98: 10541–10545. 11526221 10.1073/pnas.191362798 PMC58501

[27] Quadt-Akabayov SR, Chill JH, Levy R, Kessler N, Anglister J (2006) Determination of the human type I interferon receptor binding site on human interferon-alpha2 by cross saturation and an NMR-based model of the complex. Protein Sci 15: 2656–2668. 17001036 10.1110/ps.062283006 PMC2242419

[28] Hornak V, Abel R, Okur A, Strockbine B, Roitberg A, et al (2006) Comparison of multiple Amber force fields and development of improved protein backbone parameters. Proteins 65: 712–725. 16981200 10.1002/prot.21123 PMC4805110

[29] Yoon I, Han S, Choi YH, Kang HE, Cho HJ, et al (2012) Saturable sinusoidal uptake is rate-determining process in hepatic elimination of docetaxel in rats. Xenobiotica 42: 1110–1119. 22747239 10.3109/00498254.2012.700139

[30] Kim JE, Cho HJ, Kim JS, Shim CK, Chung SJ, et al (2013) The limited intestinal absorption via paracellular pathway is responsible for the low oral bioavailability of doxorubicin. Xenobiotica 43: 579–591. 23252722 10.3109/00498254.2012.751140

[31] Yang CH, Wu PC, Huang YB, Tsai YH (2004) A new approach for determining the stability of recombinant human epidermal growth factor by thermal Fourier transform infrared (FTIR) microspectroscopy. J Biomol Struct Dyn 22: 101–110. 15214810 10.1080/07391102.2004.10506985

[32] Dong A, Prestrelski SJ, Allison SD, Carpenter JF (1995) Infrared spectroscopic studies of lyophilization- and temperature-induced protein aggregation. J Pharm Sci 84: 415–424. 7629730 10.1002/jps.2600840407

[33] Fan H, Ralston J, Dibiase M, Faulkner E, Middaugh CR (2005) Solution behavior of IFN-beta-1a: an empirical phase diagram based approach. J Pharm Sci 94: 1893–1911. 16052555 10.1002/jps.20410

[34] Hubbard SJT (1993) NACCESS. Computer Program, Department of Biochemistry and Molecular Biology, University College London.

[35] Runkel L, Pfeffer L, Lewerenz M, Monneron D, Yang CH, et al (1998) Differences in activity between alpha and beta type I interferons explored by mutational analysis. J Biol Chem 273: 8003–8008. 9525899 10.1074/jbc.273.14.8003

[36] Runkel L, deDios C, Karpusas M, Betzenhauser M, Muldowney C, et al (2000) Systematic mutational mapping of sites on human interferon-beta-1a that are important for receptor binding and functional activity. Biochemistry 39: 2538–2551. 10704203 10.1021/bi991631c

[37] Shental-Bechor D, Levy Y (2008) Effect of glycosylation on protein folding: a close look at thermodynamic stabilization. Proc Natl Acad Sci U S A 105: 8256–8261. 18550810 10.1073/pnas.0801340105 PMC2448824

[38] Knight P (1989) The Carbohydrate Frontier. Bio-Technology 7: 35-&.

[39] Pepinsky RB, LePage DJ, Gill A, Chakraborty A, Vaidyanathan S, et al (2001) Improved pharmacokinetic properties of a polyethylene glycol-modified form of interferon-beta-1a with preserved in vitro bioactivity. J Pharmacol Exp Ther 297: 1059–1066. 11356929

[40] Wanic-Kossowska M, Tykarski A, Kobelski M, Czekalski S (2006) [Effectiveness of darbepoietin alfa in anemic patients with chronic kidney disease (CKD) in predialysis period]. Pol Arch Med Wewn 116: 663–670. 17340973

[41] Fang YW, Chang CH (2009) Subcutaneous administration of darbepoetin alfa effectively maintains hemoglobin concentrations at extended dose intervals in peritoneal dialysis patients. Perit Dial Int 29: 199–203. 19293358

[42] Walsh G, Jefferis R (2006) Post-translational modifications in the context of therapeutic proteins. Nat Biotechnol 24: 1241–1252. 17033665 10.1038/nbt1252

[43] Delorme E, Lorenzini T, Giffin J, Martin F, Jacobsen F, et al (1992) Role of glycosylation on the secretion and biological activity of erythropoietin. Biochemistry 31: 9871–9876. 1390770 10.1021/bi00156a003

