# Supplementary figures and images for: Glycoengineering of Interferon-β 1a Improves Its Biophysical and Pharmacokinetic Properties
Source: PLoS One. 2014 May 23;9(5):e96967. doi: 10.1371/journal.pone.0096967 (PMC4032242; doi:10.1371/journal.pone.0096967)

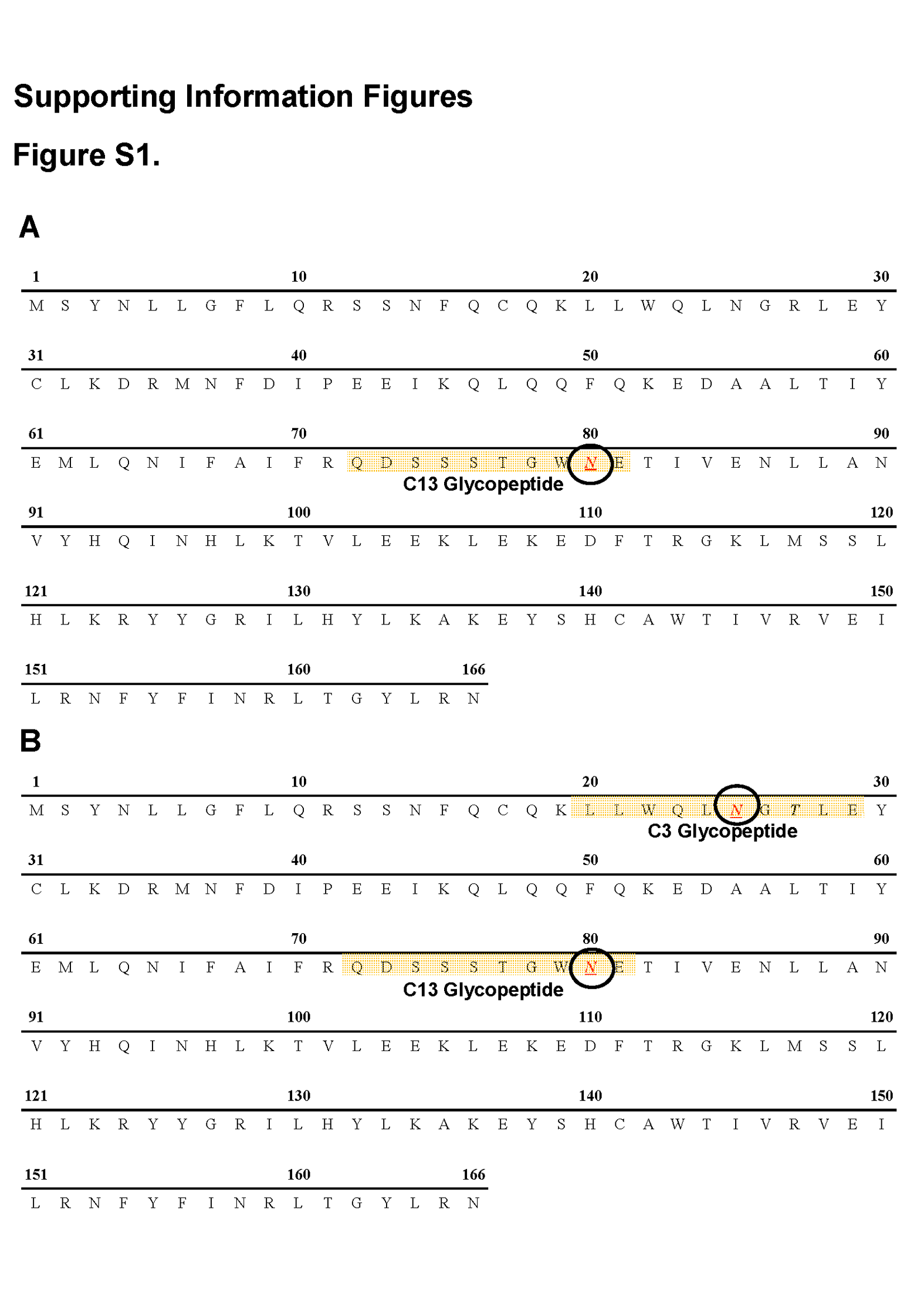

Supplement: Figure S1 — Amino acid sequence of Rebif and R27T. Shadow on amino acid showed predicted glycopeptides and circles showed potential glycosylation sites of (A) Rebif and (B) R27T. (TIF) [file pone.0096967.s001.tif]

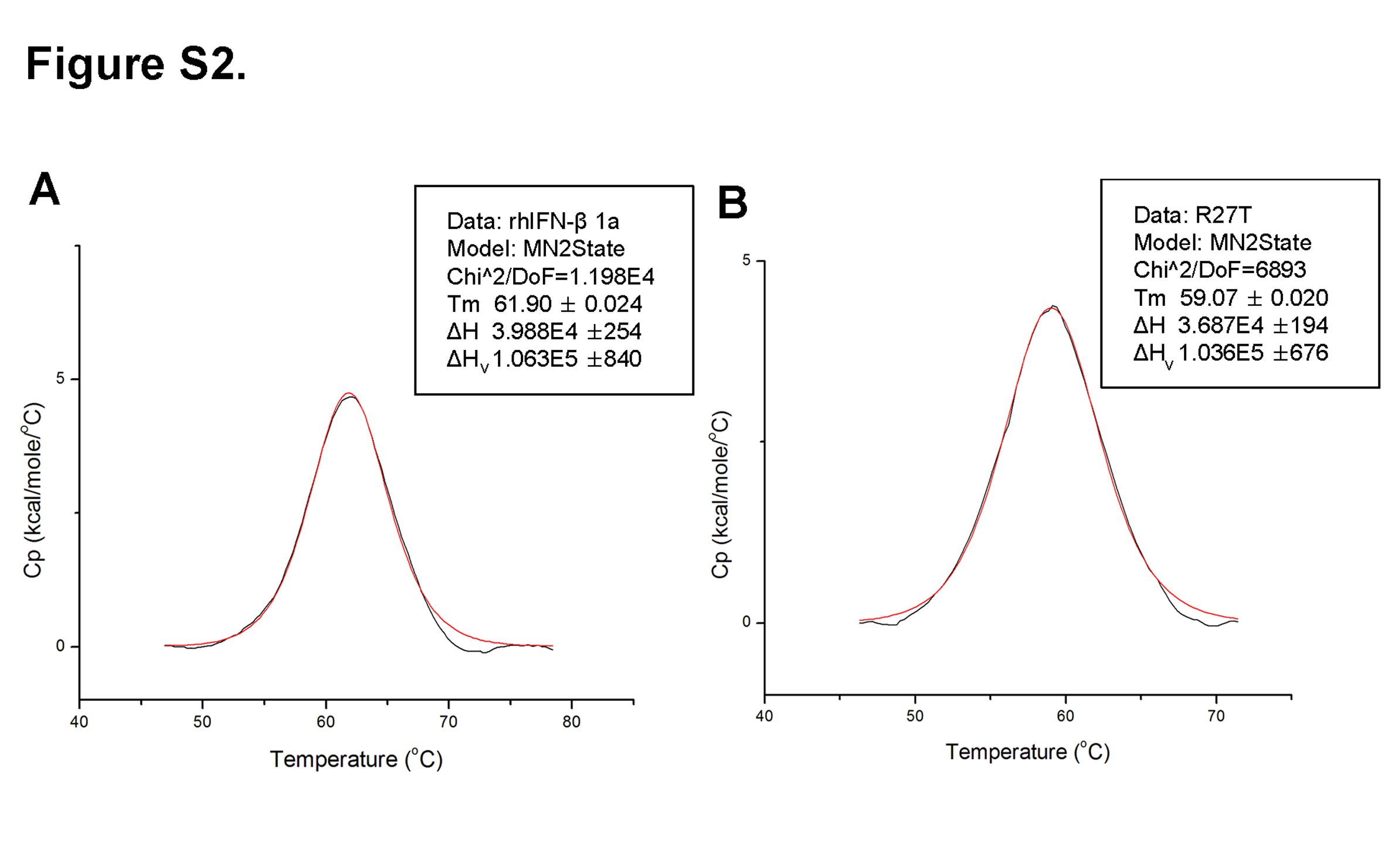

Supplement: Figure S2 — DSC thermograms of rhIFN-β 1a and R27T. Measured Tm values of (A) rhIFN-β 1a and (B) R27T was displayed above the peaks. Graphs also showed curve fitting of the DSC thermograms for rhIFN-β 1a and R27T, which was performed using a non-two-state model. (TIF) [file pone.0096967.s002.tif]
