# Supplementary material for: Glycoengineering of Interferon-β 1a Improves Its Biophysical and Pharmacokinetic Properties
Source: PLoS One. 2014 May 23;9(5):e96967. doi: 10.1371/journal.pone.0096967 (PMC4032242; doi:10.1371/journal.pone.0096967)
Supplement: Table S1 — Glycosylation site prediction. (DOCX) [file pone.0096967.s003.docx]

**Supplementary Table**

**Table S1.** Glycosylation site prediction

| # | mutation | | | substitution site | | | 80th amino acid(80N) | | | DNA sequence substitution |
| --- | --- | --- | --- | --- | --- | --- | --- | --- | --- | --- |
|  | A.A change | N site | 4 aa | potential | agree | Result | potential | agree | Result |  |
| Native | | | NETI |  |  |  | 0.5903 | 7/9 | + |  |
| 1 | **R27T** | 25N | NGTL | 0.7229 | 9/9 | ++ | 0.5902 | 7/9 | + | AGG -> ACG |
| 2 | **R27S** | 25N | NGSL | 0.6654 | 9/9 | ++ | 0.5902 | 7/9 | + |  |
| 3 | **D39T** | 37N | NFTI | 0.6983 | 9/9 | ++ | 0.5903 | 7/9 | + |  |
| 4 | **D39S** | 37N | NFSI | 0.6388 | 9/9 | ++ | 0.5902 | 7/9 | + |  |
| 5 | **Q72N** | 72N | NDSS | 0.5261 | 5/9 | + | 0.5943 | 7/9 | + |  |
| 6 | **D73N** | 73N | NSSS | 0.6810 | 9/9 | ++ | 0.5807 | 7/9 | + |  |
| 7 | **S74N** | 74N | NSST | 0.3662 | 7/9 | - | 0.5459 | 6/9 | + |  |
| 8 | **S75N** | 75N | NSTG | 0.5256 | 8/9 | + | 0.5724 | 7/9 | + |  |
| 9 | **D110N** | 110N | NFTR | 0.4982 | 2/9 | - | 0.5902 | 7/9 | + |  |
| 10 | **E137N** | 137N | NYSH | 0.5313 | 4/9 | + | 0.5902 | 7/9 | + |  |
| 11 | "+GNITVNITV" | 168N | NITV | 0.7233 | 9/9 | ++ | 0.5948 | 7/9 | + | GCTAATATCACTGTC |
|  |  | 172N | NITV | 0.6587 | 9/9 | ++ |  |  |  | AATATCACTGTC |
