# Supplementary material for: Glycoengineering of Interferon-β 1a Improves Its Biophysical and Pharmacokinetic Properties
Source: PLoS One. 2014 May 23;9(5):e96967. doi: 10.1371/journal.pone.0096967 (PMC4032242; doi:10.1371/journal.pone.0096967)
Supplement: Materials & Methods S1 — (DOCX) [file pone.0096967.s004.docx]

**Materials and Methods**

**Biophysical analysis of protein stability using Differential Scanning Calorimetry (DSC)**

DSC measurements were performed using a VP-DSC Microcalorimeter (Microcals, MA, USA). Calorimetric scans were carried out between 25 and 120˚C with a scan rate of 1˚C/ min. To determine the thermal properties of proteins in solution, heat capacity curves were generated and evaluated using Microcal Origin 7.0 software.
